# The effect of polypharmacy on rheumatoid and psoriatic arthritis treatment: retrospective study

**DOI:** 10.7717/peerj.16418

**Published:** 2023-11-22

**Authors:** Mete Kara, Gülay Alp, Seher Palanbek Yavaş, Anıl Taşdemir, Sertaç Ketenci, Müge Mercan Kara, Erkan Ozduran

**Affiliations:** 1 Rheumatology, University of Health Sciences Izmir Bozyaka Education and Research Hospital, Izmir, Turkey; 2 Rheumatology, Izmir Katip Celebi University Izmir Atatürk Education and Research Hospital, Izmir, Turkey; 3 Public Health, Canakkale Onsekiz Mart Medical Faculty, Canakkale, Turkey; 4 Internal Medicine, Izmir Bozyaka Education and Research Hospital, Izmir, Turkey; 5 Neurology, Pain Medicine, Dokuz Eylul University, Izmir, Turkey; 6 Physical Medicine and Rehabilitation, Pain Medicine, Sivas Numune Hospital, Sivas, Turkey

**Keywords:** Polypharmacy, Rheumatoid arthritis, Psoriatic arthritis, DAS-28, DMARD

## Abstract

**Background:**

Rheumatoid arthritis (RA) and psoriatic arthritis (PsA) are chronic, progressive inflammatory diseases that can be accompanied by other diseases. In recent years, with the increase in the lifespan of individuals, the concept of polypharmacy has become more prominent. We aimed to show the prevalence of polypharmacy and the effects of polypharmacy on disease activity in RA and PsA.

**Methods:**

This study included PsA patients who had peripheral joint involvement and, RA patients. Since PsA has a heterogeneous clinical picture, only patients with peripheral joint involvement were included in the study and patients with inflammatory low back pain or radiological sacroiliitis or spondylitis, dactylitis or enthesitis were not included in the study due to homogeneity concerns. The numbers of medications used by the patients at the onset of their treatment and at sixth months into their treatment were recorded. Polypharmacy was accepted as the simultaneous use of at least five medications by the person. The Disease Activity Score 28 joints C-Reactive Protein (DAS-28 CRP) was used to assess disease activity for both disease. The modified Charlson Comorbidity Index (CCI) scores of the patients were calculated based on their chronic diseases.

**Results:**

The sample of the study included 232 RA and 73 PsA patients. Polypharmacy was present at the treatment onset in 115 (49.6%) of the RA patients and 28 (38.4%) of the PsA patients. At the sixth month of treatment, polypharmacy was present in the sixth month of the treatment in 217 (93.5%) RA and 61 (83.6%) PsA patients. The mean ages of the RA and PsA patients who were receiving polypharmacy treatment at the beginning were significantly older than the mean ages of those who were not receiving polypharmacy treatment. In both the RA and PSA groups, the patients with polypharmacy at the beginning had statistically significantly higher DAS-28 CRP scores at six months of treatment than those without polypharmacy at the beginning (*p* < 0.001).

**Conclusion:**

Polypharmacy was present both at the time of diagnosis and in the treatment process in the RA and PsA patients, and the presence of polypharmacy at the beginning of the treatment was among the factors that affected the treatment of these patients by significantly affecting their 6th-month DAS-28 CRP values.

## Introduction

Rheumatoid arthritis (RA) and psoriatic arthritis (PsA) are the most common chronic and progressive inflammatory rheumatic diseases that are often accompanied by comorbidities (Smolen et al., 2018; Liu et al., 2014). Depending on the patient’s health condition, their treatment is often started as monotherapy, but it is also arranged as a combination therapy in case of unresponsiveness or presence of severe risk factors at the onset of illness (Smolen et al., 2023; Ogdie, Coates & Gladman, 2020). Additional treatments can be given to reduce the possible side effects of the treatment given in musculoskeletal diseases. Examples of this are calcium and vitamin D supplementation to reduce osteoporosis, and proton pump inhibitors used to reduce dyspeptic complaints.

Accompanying chronic diseases also increase with increased average life expectancy. Apart from the comorbidities that arise throughaging, cardiovascular and metabolic comorbidities have increased due to the diversity of drugs used in the treatment of diseases (Dougados, 2016). In RA, tumor necrosis factor inhibitors and methotrexate have been associated with reduced cardiovascular risk, while corticosteroids and non-steroidal anti-inflammatory drugs (NSAIDs) have been associated with increased risk. In PsA, there are limited data showing that systemic treatments are associated with a reduction in cardiovascular risk. As a result, the increased cardiovascular risk due to inflammatory disease decreases with treatment, and the total cardiovascular risk is a matter of debate (Roubille et al., 2015).

Over the years, the increasing prevalence of cardiovascular diseases, infections, malignancies and psychiatric diseases in patients with RA and PsA has resulted in increased comorbidity and medication use rates (Bechman et al., 2019).

In the literature, there are 143 different definitions or related terms for polypharmacy. Although different definitions are used for polypharmacy, it is commonly defined as the combined use of five or more drugs, including prescription, over-the-counter, conventional or complementary medicines (Masnoon et al., 2017). The prevalence of polypharmacy ranges from 4% among community-dwelling older people to 96.5% in hospitalized patients (Pazan & Wehling, 2021). Female sex, old age, low socioeconomic status, and increased comorbidity are associated with the frequency of polypharmacy (Filkova et al., 2017). Polypharmacy is observed in approximately half of the patients aged 65 years or older, and its prevalence has increased fourfold in the last 20 years due to the aging population and increased rates of comorbidities (Gao et al., 2018; Morin et al., 2018). A study of individuals aged >20 years in primary care in the general population showed that 16.9% of the patients used 4–9 drugs and 4.6% used g 10 drugs (Payne et al., 2014). With the increase in the prevalence of rheumatic diseases in elderly people, the concept of polypharmacy is encountered more frequently in this population (Coskun Benlidayi & Gokce Kutsal, 2022). A study on the geriatric population revealed the frequency of polypharmacy to be 62.3% (Kwan & Farrell, 2013).

Polypharmacy is one of the indicators of mortality at older ages (Jyrkkä et al., 2009). With aging, disease activity, and comorbidities increase in patients with RA and PsA (Berg et al., 1996).

Unfortunately, there are insufficient data to develop evidence-based guidelines for elderly patients, as they are often excluded from clinical trials due to age restrictions or comorbidities (Boots et al., 2013). Polypharmacy is a health condition that we do not pay much attention to, but we encounter it very often in daily practices.

The duration of the treatment is not certain in RA and PsA patients. There are a limited number of studies about the effects of polypharmacy on treatment response both at the beginning of and during the treatment. Although polypharmacy has been focused on more in recent years, a limited number of studies have been conducted in RA patients, and to the best of our knowledge, there are no published studies in PsA patients. This study aimed to present the prevalence of polypharmacy and its effect on disease activity in RA and PsA patients.

## Materials & Methods

### Ethical approval

Ethics committee approval for this study was received locally from the University of Health Sciences Izmir Bozyaka Education and Research Hospital (decision dated 13.01.2021, numbered 4). The study was conducted following the principles of the Declaration of Helsinki. Since our study was planned retrospectively, approval was obtained from the ethics committee without obtaining informed consent from the patients.

### Study design

This retrospective observational study was conducted between January 2020 and January 21 and included patients who were diagnosed to have RA according to the 2010 Rheumatoid Arthritis Classification Criteria by the American College of Rheumatology/European League Against Rheumatism (ACR/EULAR) and those who were accepted as PsA according to the classification criteria by the Classification of Psoriatic Arthritis (CASPAR) criteria (Aletaha et al., 2010; Taylor et al., 2006). Since PsA has a heterogeneous clinical picture, only patients with peripheral joint involvement were included in the study and patients with inflammatory low back pain or radiological sacroiliitis or spondylitis, dactylitis or enthesitis were not included in the study due to homogeneity concerns.

### Sample size

To evaluate polypharmacy status in rheumatoid arthritis and psoriatic arthritis patients, sample calculation was made with the G-Power (version 3.9.1.2) program. In patients with rheumatoid arthritis and psoriatic arthritis, (Bechman et al., 2019; Erdem Gürsoy et al., 2023) two independent groups exact tests were performed by predicting the rates of polypharmacy in their studies (36% and 19%, respectively), predicting the prevalence rate of rheumatoid arthritis and psoriatic arthritis as 2.5 times, alpha (*α*) margin of error as 0.05, and power as 80%. The sample size was calculated as at least 178 patients in the RA group and 71 patients in the psoriatic arthritis group, for a total of at least 249 patients.

### Demographic and clinical variables

The demographic characteristics of the patients, the number of drugs they were using, their usage of disease modiyfing anti rheumatic drugs (DMARD), smoking status, and comorbidity data were noted from the electronic patient registration system. The Disease Activity Score C-Reactive Protein (DAS-28 CRP) was used to evaluate disease activity for RA and PsA (Fransen & van Riel, 2005). Accompanying diseases were determined based ondetailed anamnesis from the patients and by examining their medical records.

The modified Charlson’s Comorbidity Index (CCI) scores were used to evaluate comorbid factors, since diseases causing comorbidities have diverse effects (Charlson et al., 1987; Beddhu et al., 2000).

Polypharmacy was defined as the simultaneous use of five or more prescription drugs. The number of drugs used by each patients was defined by the total number of different drugs prescribed and used simultaneously, excluding over-the-counter, topical and herbal/homeopathic drugs. Since our study was retrospective, these data were not included as not all of them could be fully accessed. In addition to direct information from the patients, the Social Security Board Medication Tracking System was used for the identification of polypharmacy.

The polypharmacy statuses of the patients at the onset of their treatment and at 6 months, as well as their DAS-28 CRP scores at the baseline, 6 months, and 12 months were obtained using retrospective data from the electronic patient records system. An improvement of 1.2 and above in baseline and 6th-month DAS-28 CRP scores (ΔDAS-28 CRP) was considered a major improvoment (Wells et al., 2009).

### Statistical analysis

Both visual (histogram and probability plots) and analytical methods (Kolmogorov Smirnov test) were used to check whether the variables had a normal distribution. The categorical variables are presented as percentages and frequencies, and the continuous variables are presented as mean ± standard deviation (SD) or median (IQR) values. Categorical variables were assessed by chi-square test if n ≥ 5 and Fisher’s exact test otherwise. Independent samples *t*-test was used for the comparison of two independent groups with normal distribution, and the Mann–Whitney *U* test was used for the comparisons of two groupsof non-normality distributed data. The multiple logistic regression analysis technique with the backward method was used to determine the factors associated with polypharmacy (dependent variable). Age, sex, disease duration, DAS-28 CRP, smoking status, number of comorbidities, and CCI scores were considered the independent variables. Initially, a univariate analysis was performed. The independent variables that showed statistical significance in the univariate analysis variables ( *p* < 0.20) were included in the multivariate analysis. The level of statistical significance level was accepted as *p* < 0.05. The Statistical Package for the Social Sciences (IBM SPSS) 25.0 was used for the analysis of the collected data.

## Results

### Patient characteristics

The sample of the study included a total of 232 RA and 73 PsA patients. There were 172 (74.1%) females patients in the RA group and 50 female patients (68.5%) in the PsA group. The mean age of the patients was 55.2 ± 13.66 (mean ± SD) in th RA group and 48.4 ± 13.43 (mean ± SD) in the PsA. Additionally, 140 (60.3%) of the patients in the RA group and 52 (71.2%) of the patients in the PsA group had at least one comorbidity. The mean number of drugs used by the patients at the baseline was 5.09 ± 2.89 in the RA group and 4.03 ± 2.7 in the PsA group, and the number of patients with polypharmacy at the baseline was 115 (49.6%) in the RA group and 28 (38.4%) in the PsA group. Other demographic and clinical characteristics of the patients are shown in Table 1.

**Table 1:** Demographic and clinical characteristics of RA and PsA patients.

Variables	Rheumatoid arthritis (*n* = 232)	Psoriatic arthritis (*n* = 73)
Age (mean ± SD)	55.2 ± 13.66	48.4 ± 13.43
Sex		
Female, *n* (%)	172 (74.1)	50 (68.5)
Male, *n* (%)	60 (25.9)	23 (31.5)
Age of onset, year, median (IQR )	2 (5)	1 (2)
Current smoker, *n* (%)	82 (39.2)	33 (46.5)
No smoker, *n* (%)	127 (60.8)	38 (53.5)
Number of drugs used, median (IQR)	4 (4)	4 (4)
6th month numbers of drugs used, median (IQR)	10 (6)	9 (4)
cDMARD *n* (%)	187 (80.6)	55 (75.3)
bDMARD, *n* (%)	45 (19.4)	18 (24.7)
Polypharmacy at the baseline, *n* (%)	115 (49.6)	28 (38.4)
Polypharmacy at sixth months into treatment, *n* (%)	217 (93.5)	61 (83.6)
Presence of comorbidities, *n* (%)	140 (60.3)	52 (71.2)
Number of comorbidities, median (IQR)	1 (1)	1 (1)
CCI, median (IQR)	1 (1)	1 (1)
Age Group		
<65 years, *n* (%)	171 (73.7)	65 (89)
≥65 years, *n* (%)	61 (26.3)	8 (11)
Baseline DAS-28 CRP, (mean ± SD)	4.91 ± 1.28	4.68 ± 1.03
6th-month DAS-28 CRP, (mean ± SD)	2.94 (1.64)	2.68 (1.56)
12th-month DAS-28 CRP (*n* = 83/29),median (IQR)	2.45 (1.6)	2.47 (1.91)
Decrease in DAS-28 CRP scores from the baseline to the 6th month	1.8 (2.02)	1.84 (1.44)

**Notes.**

RA Rheumatoid arthritis PsA Psoriatic arthritis IQR Interquartile Range DAS-28 CRP Disease Activity Score-28 C-Reactive Protein CCI Modified Charlson Comorbidity Index cDMARD conventional disease-modifying antirheumatic drug bDMARD biologic disease modifying anti-rheumatic drug DMARD Disease Modifying Anti-Rheumatic Drugs

### Polypharmacy

#### Comparison of the patients based on the presence of polypharmacy at the baseline

The rate of decrease in DAS-28 CRP scores at the 6th month of the treatment in RA and PsA patients (1.26 ± 1.28 in those with polypharmacy and 2.25 ± 1.28 in those without polypharmacy) was significantly different from the rate of decrease in their DAS-28 CRP scores at the 12th month in the treatment (1.44 ± 1.65 in those with polypharmacy, 2.64 ± 1.44 in those without) (*p* < 0.001 and *p* < 0.001).

In the RA group, the baseline DAS-28 CRP score was 5.08 ± 1.3 for those without baseline polypharmacy and 4.74 ± 1.25 for those with baseline polypharmacy, and these values were significantly different from each other (*p* = 0.046). In the PsA group, there was no significant difference between the baseline DAS-28 CRP scores of those with and without polypharmacy at the baseline (*p* = 0.3). In both the RA and PsA groups, the DAS-28 CRP scores at the 6th month of treatment were higher in those with baseline polypharmacy than in those without baseline polypharmacy (*p* < 0.001). The significant association between the high DAS-28 CRP scores and having baseline polypharmacy in the RA group persisted at the 12th month of treatment (*p* = 0.006), while this association was no longer significant for those in the PSA group (*p* = 0.07).

The proportion of major improvement (an improvement in DAS-28 CRP score of 1.2 or above) was 36.5% for the patients with baseline polypharmacy and 63.5% for those without baseline polypharmacy. A statistically significant difference was found between these two groups (*p* < 0.001). Considering the RA and PsA patients with baseline polypharmacy separately, the rate of major improvement was found to be significantly lower in those with polypharmacy (*p* < 0.001 and *p* = 0.004, respectively). Table 2 presents the characteristics of the RA and PsA patients with and without baseline polypharmacy.

**Table 2:** Comparison of rheumatoid and psoriatic arthritis patients with and without polypharmacy at baseline.

	RA, without polypharmacy (*n* = 117)	RA, with polypharmacy (*n* = 115)	*p*	PsA, without polypharmacy (*n* = 45)	PsA, with polypharmacy (*n* = 28)	*p*
Age, (mean ± SD), y	51.2 ± 13.5	59.2 ± 12.6	**<0.001**	45.2 ± 12.3	53.5 ± 13.8	**0.009**
≥65 years vs <65 years, *n* (%)	20 (32.8)	41(67.2)	**0.001**	2 (25)	6 (75)	**0.048**
Sex, female, *n* (%)	86 (50)	86 (50)	0.824	33 (66)	17 (34)	0.259
Age of onset, median (IQR), y	2 (5)	2 (7)	0.232	1 (3)	1 (2)	0.824
Presence of comorbidities *n* (%)	55 (39.3)	85 (60.7)	**<0.001**	31 (59.6)	2 (40.4)	0.575
Number of comorbidities, median (IQR)	0 (1)	1 (2)	**<0.001**	1 (1)	1 (1)	0.166
CCI, median (IQR)	1 (0)	1 (1)	**<0.001**	1 (0)	1 (1)	**<0.001**
Smoking, *n* (%)	38 (46.3)	44 (53.7)	0.259	18 (54.5)	15 (45.5)	0.150
DAS-28 CRP at the baseline, (mean ± SD)	5.08 ± 1.3	4.74 ± 1.25	**0.046**	4.58 ± 1.02	4.84 ± 1.05	0.3
DAS-28 CRP at the 6th month, (mean ± SD)	2.8 ± 0.86	3.48 ± 1.13	**<0.001**	2.41 ± 0.54	3.59 ± 1.13	**<0.001**
DAS-28 CRP at the 12th month median (IQR) (*n* = 35/48)	1.91 (1.36)	2.62 (1.77)	**0.006**	2.07 (1.04)	3.14 (2.36)	0.07^a^
ΔDAS-28 CRP*, *n* (%)	91 (60.7)	59 (39.3)	**<0.001**	38 (71.7)	15 (28.3)	**0.004**
Number use of drugs in baseline, median (IQR)	3 (1)	7 (4)	**<0.001**	3 (2)	6 (2)	**<0.001**
Number of drugs used at the 6th month median (IQR)	7 (3)	12 (4)	**<0.001**	7 (4)	10 (4)	**<0.001**
cDMARD, *n* (%)	97 (56.1)	76 (43.9)	**0.003**	34 (63)	20 (37)	0.696
b/tsDMARD, *n* (%)	20 (33.9)	39 (66.1)		11 (57.9)	8 (42.1)	
Single DMARD, *n* (%)	36 (63.2)	21 (36.8)	0.188	22 (62.9)	13 (37.1)	0.983
Combined DMARD, *n* (%)	61 (52.6)	55 (47.4)		12 (63.2)	7 (36.8)	

**Notes.**

ΔDAS-28 CRP >1.2 changes in baseline and 6.-month DAS-28 CRP scores RA Rheumatoid arthritis y years IQR Interquartile Range DAS 28 CRP Disease Activity Score-28 C-Reactive Protein cDMARD conventional disease-modifyingantirheumatic drug bDMARD biologic disease modifying anti-rheumatic drug tsDMARD targeted synthetic disease modifying anti-rheumatic drugs DMARD Disease Modifying Anti-Rheumatic Drugs

a Exact significance is not, bold values denote statistical significance at the *p* < 0.05 level.

When categorized as number of drugs used <10 and g10, the 6th-month median DAS-28 CRP scores were 2.83 (1.69) and 3.81 (1.82) in the RA patients with and without polypharmacy, and their 12th-month DAS-28 CRP scores were 2.4 (1.42) and 4.76 (2.92), respectively; where the differences between the groups created based on the numbers of drugs the patients used were statistically significant difference was found between them (*p* = 0.044 and 0.012, respectively). A statistical analysis could not be performed for the PsA patients because of the low number of those whose used 10 or more drugs.

### Comparison of the patients based on the presence of polypharmacy at the 6th month of treatment

In the RA group, the mean baseline DAS-28 CRP score of the patients who had polypharmacy at the 6th month was 4.66 ± 1.45 and the mean baseline score of those without polypharmacy at the 6th month was 4.93 ± 1.27.

In the PsA group, the mean baseline DAS-28 CRP score of the patient who had polypharmacy at the 6th month was 4.34 ± 8 and the mean baseline score of those without polypharmacy at the 6th month was 4.75 ± 1.06.

There was no statistically significant difference between the baseline DAS-28 CRP scores of those who had polypharmacy at the 6th month of treatment and those who did not have polypharmacy at the 6th month in either groups (*p* = 0.43 and *p* = 0.211, respectively). In the intra-group comparisons of the RA and PsA groups, 6th-month DAS-28 CRP scores were found to be statistically significantly higher in those with polypharmacy at the 6th month of treatment than in those without polypharmacy at the 6th month of treatment (*p* = 0.048 and *p* = 0.005, respectively). The significant association between the high DAS-28 CRP scores and having polypharmacy at the 6th month of treatment in the RA group continued at the 12th month of treatment (*p* = 0.042), while this association was no longer significant for those in the PSA group (*p* = 0.206). Table 3 presents the characteristics of the RA and PsA patients with and without polypharmacy at the 6th month of treatment.

**Table 3:** Comparison of rheumatoid and psoriatic arthritis patients with and without polypharmacy at six months.

	**RA,** **without polypharmacy** **(*n* = 15)**	**RA,** **with polypharmacy** **(*n* = 217)**	*p*	**PSA,** **without polypharmacy** **(*n* = 45)**	**PSA,** **with polypharmacy** **(*n* = 28)**	*p*
Age (mean ± SD), y	50 ± 16.8	55.5 ± 13.9	0.128	38.3 ± 12.3	50.3 ± 12.8	**0.004**
Age ≥65 years, *n* (%)	3 (4.9)	58 (95.1)	0.765	0 (0)	8 (100)	0.338
Sex, Female, *n* (%)	11 (6.4)	161 (93.6)	1.000	8 (16)	42 (84)	1.000
Age of onset, y median (IQR),	2 (3)	2 (6)	0.66	1 (1)	1 (3)	0.052
Presence of comorbidities, *n* (%)	7 (5)	133 (95)	0.263	5 (9.6)	47 (90.4)	**0.031**
Number of comorbidities, median (IQR)	0 (0–1)	1 (0–2)	0.095	0 (0–1)	1 (1–1)	**0.022**
CCI, median (IQR)	0 (1)	1 (1)	**<0.001**	1 (0)	1 (1)	**0.026**
Smoking, *n* (%)	4 (4.9)	78 (95.1)	0.519	3 (9.1)	30 (90.9)	0.123
DAS-28 CRP at the baseline (mean ± SD)	4.66 ± 1.45	4.93 ± 1.27	0.43	4.34 ± 0.83	4.75 ± 1.06	0.211
DAS-28 CRP at the 6th.month, (mean ± SD)	1.74 (2.07–2.58)	2.22(2.8–3.38)	**0.048**	2.07 (1.74–2.58)	2.8 (2.22–3.38)	**0.005**
DAS-28 CRP the 12th month, median (IQR)	1.5 (1.4–)	2.55 (1.8–3.36)	**0.042**	3.47 (2.22–4.41)	2.3 (1.83–3.76)	0.206
cDMARD	11 (6.4)	162 (93.6)	1	10 (18.5)	44 (81.5)	0.720
bDMARD	4 (6.8)	55 (93.2)		2 (10.5)	17 (89.5)	
Single DMARD, *n* (%)	7 (12.3)	50 (87.7)	**0.042**	7 (20)	28 (80)	1
Combined DMARD, *n* (%) 4 (3.4)	112 (96.6)		3 (15.8)	16 (84.2)	
bDMARD/tsDMARD at the baseline *n* (%)	20 (33.9)	39 (66.1)	**0.003**	11 (57.9)	8 (42.1)	0.696

**Notes.**

RA Rheumatoid arthritis y years IQR Interquartile Range DAS 28 CRP Disease Activity Score-28 C-Reactive Protein cDMARD conventional disease-modifying antirheumatic drug bDMARD biologic disease modifying anti-rheumatic drug tsDMARD targeted synthetic disease modifying anti-rheumatic drugs DMARD Disease Modifying Anti-Rheumatic Drugs CCI Modifiedthe Charlson Comorbidity Index

Bold values denote statistical significance at the *p* < 0.05 level.

Age, the presence of comorbidities, biological DMARD (bDMARD) use, and baseline DAS-28 CRP scores were found to be significantly effective variables in the univariate regression analysis in which the factors affecting the presence of polypharmacy at the baseline were evaluated for patients in the RA group. These variables were evaluated together in the multiple regression analysis in model 1, where age, number of comorbidities, baseline DAS-28 CRP scores and bDMARD use were found to be independently associated with the presence of polypharmacy. In the model 2, an age 65 years or older, the presence of acomorbidity, baseline DAS-28 CRP score and bDMARD use were found to be independently associated with the presence of polypharmacy (Table 4).

**Table 4:** Evaluation of the factors affecting the presence of polypharmacy in RA patients at the baseline.

	RA univariate analysis		RA multiple analysis model-1		RA multiple analysis model-2	
Variables	**OR (** **95% CI)**	*p*	**OR (** **95% CI)**	*p*	**OR** **(** **95% CI)**	*p*
Age	1.047 (1.025–1.070)	**<0.001**	1.037 (1.013–1.061)	**0.002**		
Age ≥65 years	0.372 (0.201–0.688)	**0.002**			0.309 (0.134–0.713)	**0.006**
Sex, Female	1.069 (0.594–1.925)	0.824				
Age of onset	1.038 (0.996–1.082)	0.078				
Smoking	0.726 (0.416–1.267)	0.260				
Presence of comorbidities	0.313 (0.180–0.544)	**<0.001**			0.391 (0.191–0.798)	**0.010**
Number of comorbidities	1.970 (1.494–2.597)	**<0.001**	1.691 (1.260–2.269)	**<0.001**		
CCI	2.725 (1.834–4.048)	**<0.001**				
Baseline DAS-28 CRP	0.813 (0.662–0.998)	**0.048**	0.654 (0.508–0.843)	**0.001**	0.643 (0.486–0.848)	**0.002**
bDMARD use	0.402 (0.217–0.745)	**0.004**	0.297 (0.144–0.614)	**0.001**	0.326 (0.149–0.713)	**0.005**

**Notes.**

RA Rheumatid arthritis DAS- 28 CRP Disease Activity Score-28 C-Reactive Protein bDMARD biologic disease modifying anti-rheumatic drug DMARD Disease Modifying Anti-Rheumatic Drugs CCI the Charlson Comorbidity Index OR Odds Ratio

Bold values denote statistical significance at the *p* < 0.05 level.

Age and CCI were determined to be significantly effective variables in the univariate regression analysis in which the factors affecting the presence of polypharmacy at the baseline were evaluated for the patients in the PsA group.

These variables were evaluated together in the multiple regression analysis of model 1 and only CCI was found to be independently associated with the presence of polypharmacy. When the age variable was categorized as an age 65 years or older included in the multiple regression analysis of model 2, age and CCI were revealed to be independently associated with the presence of polypharmacy (Table 5).

**Table 5:** Evaluation of the factors affecting the presence of polypharmacy in psoriatic arthritis patients at baseline.

	**PsA univariate analysis**		**PsA multiple analysis** **model 1**		**PsA multiple analysis model 2**	
**Variables**	**OR (** **95% CI)**	*p*	**OR (** **95% CI)**	*p*	**OR (** **95% CI)**	*p*
Age	1.051 (1.010–1.094)	**0.014**	1.037 (0.994–1.083)	0.092		
Age ≥65 years	0.171 (0.032–0.916)	**0.039**			0.138 (0.023–0.825)	**0.03**
Sex, Female	0.562 (0.206–1.537)	0.261				
Age of onset	0.947 (0.841–1.066)	0.364				
Smoking	0.489 (0.183–1.303)	0.152				
Presence of comorbidities	0.738 (0.255–2.137)	0.576				
Number of comorbidities	1.742 (0.972–3.120)	0.062				
CCI	5.627 (1.884–16.807)	**0.002**	4.780 (1.587–14.399)	**0.005**	6.497 (2.048–20.615)	**0.001**
Baseline DAS-28 CRP	1.282 (0.803–2.047)	0.298				
bDMARD use	0.809 (0.279–2.347)	0.696				

**Notes.**

PsA Psoriatic arthritis DAS 28 CRP Disease Activity Score-28,C Reactive Protein bDMARD biologic disease modifying anti-rheumatic drug DMARD Disease Modifying Anti-Rheumatic Drugs CCI Modified Charlson Comorbidity Index

Bold values denote statistical significance at the *p* < 0.05 level.

Age and CCI were determined to be significantly effective variables in the univariate regression analysis in which the factors affecting the presence of polypharmacy at the 6th month of treatment were evaluated for the patients in the RA group. These variables were evaluated together in the multiple regression analysis, and only CCI (OR, 3.206, 95% CI [1.270–8.092], *p* = 0.014) was found to be independently associated with the presence of polypharmacy. Age, number of additional diseases and 6th-month DAS-28 CRP score were found to be significant in the univariateregression analysis in which the factors affecting the presence of polypharmacy at the 6th month of treatment were evaluated for the patients in the PsA group. These variables were evaluated together in the multiple regression analysis, and only 6th-month DAS-28 CRP score (OR, 4.742, 95% CI [1.246–18.04], *p* = 0.022) were found to be independently associated with the presence of polypharmacy (Table 6).

**Table 6:** Evaluation of the factors affecting the presence of polypharmacy in rheumatoid arthritis and psoriatic arthritis at sixth months.

	**RA univariate analysis**		**RA multiple analysis**		**PsA univariate analysis**		**PsA multiple analysis**	
**Variables**	**OR** **(** **95% CI)**	*p*	**OR** **(** **95% CI)**	*p*	**OR** **(95%** **CI)**	*p*	**OR** **(95%** **CI)**	*p*
Age	1.029 (0.991–1.069)	**0.130**	1.006 (0.963–1.052)	0.781	1.086 (1.022–1.155)	**0.008**	1.519 (0.746–3.092)	0.249
Sex, Female	0.957 (0.293–3.126)	0.941			0.905 (0.242–3.376)	0.882		
Age of onset	1.032 (0.935–1.138)	0.534			1.793 (0.829–3.877)	0.138		
Smoking	0.672 (0.200–2.259)	0.521			0.322 (0.079–1.310)	0.114		
Presence of comorbidities	0.553 (0.193–1.580)	0.269			0.213 (0.058–0.776)	**0.019**		
Number of comorbidities					3.057 (1.067–8.753)	**0.037**	2.296 (0.759–6.942)	0.141
CCI	3.350 (1.400–8.016)	**0.007**	3.206 (1.270–8.092)	**0.014**	3.713 (0.745–18.499)	0.109		
Baseline DAS-28 CRP	1.182 (0.781–1.789)	0.429			1.535 (0.785–3.000)	0.210		
6th. month DAS-28 CRP	1.428 (0.813–2.511)	**0.215**			4.301 (1.334–13.866)	**0.015**	4.742 (1.246–18.046)	**0.022**
ΔDAS-28 CRP	0.959 (0.656–1.401)	0.827			0.743 (0.429–1.288)	0.290		
bDMARD use	1.071 (0.328–3.502)	0.910			0.518 (0.103–2.611)	0.425		

**Notes.**

RA Rheumatoid arthritis PsA Psoriatic arthritis DAS- 28 CRP Disease Activity Score-28 C-Reactive Protein bDMARD biologic disease modifying anti-rheumatic drug DMARD Disease Modifying Anti-Rheumatic Drugs ΔDAS-28 CRP The rate of improvement in DAS-28 CRP score by 1.2 and above

Bold values denote statistical significance at the *p* < 0.05 level.

## Discussion

To the best of our knowledge, this is the first study to evaluate how polypharmacy affects disease activity at the beginning, sixth month and twelfth months of treatment in patients with RA and PsA and examine other factors associated with polypharmacy in these patients. Although there have been awareness and studies about polypharmacy in recent years, studies about its effects on treatment response in RA and PsA patients are very limited (Kara et al., 2022). In our study, the presence of polypharmacy at the baseline in RA and PsA patients was found to be associated with a higher DAS-28 CRP score at the 6th month of treatment. This relationship persisted at the 12th month of treatment among the RA patients, but while it could no longer observed in the PsA patients due to the small sample size of the group. Likewise, the rates of major improvement at the 6th month of treatment were found to be higher in the patients without polypharmacy in both groups.

In rheumatology, studies on polypharmacy are mostly conducted on older adults, monitoring side effects and comorbidities. The effects of polypharmacy on treatment response in rheumatological diseases were first reported in 2019 by the British Society for Rheumatology Biologics Register for Rheumatoid Arthritis (BSRBR-RA). In the study, polypharmacy was accepted as the use of 6 or more drugs and was detected in 36% of RA patients. In studies conducted with RA patients, the prevalence of polypharmacy was found to be 64.5%–67.9% (Gomides et al., 2021; Ma, Zaman Huri & Yahya, 2019). The results of our study revealed the frequency of polypharmacy in the RA patients before treatment as 49.6%, which was consistent with other rates reported in the relevant literature.

The prevalence of comorbidities and consequently polypharmacy increases by aging and may lead to treatment failure. The study conducted by the BSRBR-RA evaluated comorbidities as one of the factors related to polypharmacy and reported that the use of an additional drug due to a comorbidity reduced the degree of response to treatment by 8%. The aforementioned study showed that the more drugs patients toke in addition to those in their rheumatic disease treatment, the less likely they were to achieve clinically significant disease improvement. Our study demonstared that the presence of polypharmacy at the baseline negatively affected the decrease in disease activity at sixth months into the treatment in both the RA and PsA patients.

In our study, while the rate of baseline polypharmacy was 49.6% and 38.4% in the RA and PsA groups, respectively; this rate increased to 93.5% and 83.6% at the sixth month of treatment in these respective groups. This may be because of the treatment characteristics of rheumatic disease. In addition to rheumatic treatments, drugs given for the treatment or prevention of osteoporosis and gastrointestinal system issues also contribute to polypharmacy. Our results highlighted that baseline polypharmacy was independently associated with age, number of additional diseases, baseline DAS-28 CRP scores, and bDMARD use in the RA patients and aged 65 or above and CCI scores in PsA the patients. Additionally, polypharmacy at the 6th month of treatment was independently associated with CCI scores in the RA patients and 6th month DAS-28 CRP scores in the PsA patients.

A study reported that bDMARDs that are not metabolized by cytochrome P450 or cleared by renal elimination may be less affected by polypharmacy (Bechman et al., 2019). Our study showed that the presence of baseline polypharmacy was high in the RA patients (*p* = 0.003) in terms of starting bDMARD in the follow-up period, while it was not statistically significantly high in the PsA patients (*p* = 0.696). This may be due to the inclusion of fewer patients in the PsA group, in which fewer patients Were reveiving treatment with bDMARDs. A study on the factors associated with polypharmacy in RA patients reported a higher usage rate of bDMARDs in those with polypharmacy (Jack et al., 2020). In our study, we determined that the rate of polypharmacy was higher in the RA and PsA patients over 65 years of age compared to those under 65 years of age (*p* = 0.001 and *p* = 0.048, respectively). However, cDMARD and bDMARD usage rates were similar between these two groups. In the treatment of elderly patients, pharmacotherapy modalities differ due to polypharmacy (Juby & Davis, 2011). Elderly patients need more treatments due to comorbidities, are at a higher risk of polypharmacy, and therefore, they need to be taken care of in this regard.

A survey study on the factors affecting treatment choice by rheumatologists in RA patients Revealed that patient age and deterioration had similar effects on treatment choice, following their DAS scores (Kievit et al., 2010). While the aforementioned study showed that rheumatologists were less willing to change the treatment of elderly patients, another study reported that younger RA patients were more likely to prefer aggressive DMARD therapy than older patients (Fraenkel, Rabidou & Dhar, 2006). Furthermore, another study demonstrated that comorbidities and polypharmacy were significantly high in RA patients with drug-related problems (Ma, Zaman Huri & Yahya, 2019). Comorbidities have also been shown to affect adherence to treatment in patients with RA (Murage et al., 2018). As the patient’s age increases, their probability of having comorbidity and polypharmacy also increases, and rheumatologists should pay attention to all these conditions in their treatment decisions.

In a previous study, the prevalence of polypharmacy was found as 69.5% in RA patients and the mean number of drugs used by these patients was 5.39 (maximum 16), of which an mean of 2.41 drugs were directly for the treatment of RA; and the authors reported that this condition was associated with age, duration of disease, and comorbidity status (Treharne et al., 2007). Unlike our study, Treharne et al. (2007) found that polypharmacy was not associated with disease activity. We observed that in both RA and PsA groups, the 6th mean DAS-28 CRP scores of those without baseline polypharmacy were statistically significantly lower than the scores of those with baseline polypharmacy (*p* < 0.001). As our study was designed to compare the therapeutic effects of pre-treatment polypharmacy, likely to determine a healthier association with disease activity.

There is increased comorbidity in PsA and RA patients (Sinnathurai et al., 2018). Two individuals with the same disease may not be affected to the same extent by the disease. The use of a single or combined medication for a disease can provide us with different information about the severity of that disease. Polypharmacy may provide us with more insight than routine comorbidity indices. In our study, we found that CCI scores were significantly higher in both the RA and PsA patients at the baseline and in the RA patients with polypharmacy only at the 6th month of treatment. This had no significant effect on ΔDAS-28 CRP (>1.2 change in baseline and 6th-month DAS-28 CRP score) (*p* = 0.066).

PsA is a heterogeneous inflammatory disease with many different clinical phenotypes associated with psoriasis. Epidemiological studies have shown the presence of several comorbidities that can lead to increased mortality and morbidity rates in PsA patients (Woo et al., 2020; Erdem Gürsoy et al., 2023). Since additional treatment is usually required for comorbidities, patients with comorbidities are expected to have higher rates of polypharmacy compared to patients who do not.

The frequency of polypharmacy in PsA patients was determined to be 19% when the number of drugs they used was ≥ 5, and 59% when the number of drugs they used was 2–4 (Erdem Gürsoy et al., 2023). In our study, the frequency of polypharmacy in the PsA patients was 39.4% at the beginning of treatment and 83.6% at the 6th-month of treatment. The aforementioned study found no significant relationship between polypharmacy and disease activity, however, our study revealed that the presence of baseline polypharmacy was associated with significantly higher DAS-28 CRP scores at the 6th month of treatment (*p* < 0.001). Our study is the first study in the literature to show that polypharmacy has an effect on treatment in PsA patients.

There are few studies on the relationship between comorbidities and polypharmacy in PsA patients. These studies have shown the relationships among polypharmacy, comorbidity burden, low quality of life, and anxiety (Gupta et al, 2021).

A study on compliance with bDMARD in PsA patients reported the factors increasing drug compliance as non-use of steroids and NSAIDs and low comorbidity burden levels (Vangeli et al., 2015). The presence of both extra-articular involvement and comorbidities in PsA patients may have a negative impact on prognosis and treatment response (Haroon & FitzGerald, 2016).

### Strengths and limitations

As one of its strengths, our study reflects the data of patients in daily practice, better compared to controlled studies with selected groups of young patients with low comorbidity rates.

On the other hand, one of the limitations of our study was that it was designed retrospectively and only prescription drugs could be recorded, excluding over-the-counter medications or functional or complementary medicine treatments. In our study, prescription drugs were not specified individually. The drugs used were not divided into groups, and the effects of anti-rheumatic treatments or other treatment groups were not clearly evaluated. Apart from this, the participating centers were tertiary/university hospitals, and therefore, the patients were likely in a more severe condition.

## Conclusion

In conclusion, polypharmacy is a very common adverse condition in RA and PsA patients, affecting disease activity. In our study, polypharmacy was present in the RA and PsA patients both at the time of diagnosis and during the treatment process, and the presence of baseline polypharmacy was among the factors that affected the treatment of these patients by significantly affecting their 6th-month DAS-28 CRP values. Polypharmacy should be considered as one of the factors affecting the treatment of patients with RA and PsA, which are chronic inflammatory arthritis, and should be taken into account when individualizing their treatment.

## Supplemental Information

10.7717/peerj.16418/supp-1 Supplemental Information 1 Raw Data (SPSS)

## Additional Information and Declarations

### Data Availability

The following information was supplied regarding data availability: The raw measurements are available in the Supplementary File.
